# DNA Damage Repair in Brain Tumor Immunotherapy

**DOI:** 10.3389/fimmu.2021.829268

**Published:** 2022-01-13

**Authors:** Shihong Zhao, Boya Xu, Wenbin Ma, Hao Chen, Chuanlu Jiang, Jinquan Cai, Xiangqi Meng

**Affiliations:** Department of Neurosurgery, The Second Affiliated Hospital of Harbin Medical University, Harbin, China

**Keywords:** DNA damage repair, brain tumor, immunotherapy, TME, TAMs, ICI, biomarker, combination therapy

## Abstract

With the gradual understanding of tumor development, many tumor therapies have been invented and applied in clinical work, and immunotherapy has been widely concerned as an emerging hot topic in the last decade. It is worth noting that immunotherapy is nowadays applied under too harsh conditions, and many tumors are defined as “cold tumors” that are not sensitive to immunotherapy, and brain tumors are typical of them. However, there is much evidence that suggests a link between DNA damage repair mechanisms and immunotherapy. This may be a breakthrough for the application of immunotherapy in brain tumors. Therefore, in this review, first, we will describe the common pathways of DNA damage repair. Second, we will focus on immunotherapy and analyze the mechanisms of DNA damage repair involved in the immune process. Third, we will review biomarkers that have been or may be used to evaluate immunotherapy for brain tumors, such as TAMs, RPA, and other molecules that may provide a precursor assessment for the rational implementation of immunotherapy for brain tumors. Finally, we will discuss the rational combination of immunotherapy with other therapeutic approaches that have an impact on the DNA damage repair process in order to open new pathways for the application of immunotherapy in brain tumors, to maximize the effect of immunotherapy on DNA damage repair mechanisms, and to provide ideas and guidance for immunotherapy in brain tumors.

## Introduction

DNA is a nucleic acid that is a key material essential for the body to function properly. However, DNA is affected by various endogenous and exogenous factors every day, such as ionizing radiation, benzene, Epstein-Barr virus and other physical, chemical and biological factors (1), and internal due to the activation of proto-oncogenes (2). Fortunately, the body has a variety of DNA repair pathways to correct and repair the damaged location in a timely manner to ensure the stability and integrity of the eukaryotic genome. There are currently known DNA damage repair pathways, namely: mismatch repair (MMR), base excision repair (BER), nucleotide excision repair (NER), homologous recombination repair (HRR), nonhomologous end-joining (NHEJ). Among them, both HRR and NHEJ are used to repair double strand breaks (DSBs) (3). Once the damage mechanism is dysregulated, the stability of the genome will be disrupted and cells will suffer various damages such as inflammation, aging and even cancer (4, 5). For example, MMR defects can increase the risk of hereditary colon cancer (6), and BER defects can increase the risk of lung cancer (7). Meanwhile, the exploration of tumor immunotherapy is rapidly evolving. Unlike traditional tumor treatments, tumor immunotherapy aims to restore the body’s normal anti-tumor immune response by initiating the tumor immune cycle to accomplish the recognition and clearance of tumor cells (8). This property allows immunotherapy to maintain longer survival or even achieve clinical cure by the body’s own action once the immune system in the body is awakened. Currently, the following immunologic agents are approved by the FDA and used in clinical work (**Table 1**). However, immunotherapy is extremely selective in terms of target populations and not all tumor patients benefit from immunotherapy. For example, brain tumors are a class of “cold tumors” in the immunological sense. Due to their unique tumor immune microenvironment (TME), they are the least sensitive to immunotherapy. Currently, surgery, radiation and chemotherapy are still widely accepted in brain tumors. Meanwhile, neurorestorative treatment have also emerged in recent years and have been shown to repair the function of damaged nerves in some sense (9). For example, olfactory ensheathing cells, with their superior ability to integrate and repair nerves (10, 11), interact with glial scars to stimulate vascular and axonal growth (12), in order to repair brain nerves after tumor damage and help restore brain function (13). In contrast to these rapid developments, immunotherapy, which has achieved superior therapeutic results in other tumors, remains ineffective in brain tumors. But, since tumor cells also depend on DNA damage repair pathways for their survival and reproduction as normal cells do (14), then rational disruption of the DNA damage repair pathways of tumor cells is a major way to overcome tumors. This mechanism has been used in brain tumor cell therapy for a long time, and many radiotherapy and chemotherapy treatments are based on it. Then, the use of DNA damage repair mechanism to improve the effect of brain tumor immunotherapy is a breakthrough, and whether this mechanism can be used to complete the immunotherapy of “cold tumors” is the focus of this paper. The purpose of this paper is to discuss this idea in the following four aspects. First, we review the specific process of DNA damage repair and identify key site that may influence immunotherapy. Second, the repair mechanisms involved in the action of the most common immunotherapy, immune checkpoint inhibitors, will be described. Subsequently, we will screen existing or potential site from the DNA damage repair process, assess their place in brain tumor immunotherapy, and discuss their feasibility and predictiveness as biomarkers for brain tumor immunotherapy. Finally, based on the process of DNA damage repair, it is reasonable to think about the feasibility of combining immunotherapy with other therapeutic approaches applying this principle, which is currently a hot research direction for the rational inclusion of immunotherapy in the brain tumor population. To explore the place of DNA damage repair in immunotherapy of brain tumors and its broad development prospect, and to provide some clinical guidance.

**Table 1:** FDA-approved immunotherapies.

Immunotherapy category	Mechanism	Therapy	FDA-approved cancers	Year of approval
Checkpoint inhibitors	Anti-CTLA-4	Ipilimumab	Melanoma	2011
			Advanced renal cell carcinoma	2018
			MSI-H/dMMR metastatic colorectal cancer	
			Hepatocellular carcinoma(HCC)	2020
			Metastatic or recurrent non-small cell lung cancer	
			Malignant pleural mesothelioma	
	Anti-PD-1	Pembrolizumab	Melanoma	2014
			Non-small cell lung cancer(NSCLC)	2015
			Head and neck squamous cell carcinoma(HNSCC)	
			Classical Hodgkin lymphoma(CHL)	2017
			Advanced or metastatic urothelial carcinoma	
			MSI-H/dMMR solid tumors	
			Advanced gastric cancer	
			Recurrent or metastatic cervical cancer	2018
			Primary mediastinal large B-cell lymphoma (PMBCL)	
			Advanced or metastatic Merkel cell carcinoma(MCC)	
			Advanced renal cell carcinoma(RCC)	2019
			Esophageal cancer	
			Hepatocellular carcinoma(HCC)	
			Endometrial carcinoma	
			Metastatic small cell lung cancer (SCLC)	
			MSI-H/dMMR metastatic colorectal cancer	2020
			Tumor mutational burden-high (TMB-H) solid tumors	
			Non-muscle invasive bladder cancer (NMIBC)	
			Recurrent or metastatic cutaneous squamous cell carcinoma (cSCC)	
			Advanced esophageal or gastroesophageal (GEJ) carcinoma	2021
			Triple-negative breast cancer (TNBC)	
		Nivolumab	Melanoma	2014
			Non-small-cell lung cancer	2015
			Renal cell carcinoma	
			Classical Hodgkin lymphoma(cHL)	2016
			Head and neck squamous cell carcinoma	
			Urothelial carcinoma(UC)	2017
			MSI-H/dMMR colorectal cancer	
			Hepatocellular carcinoma(HCC)	
			Metastatic small cell lung cancer(SCLC)	2018
			Advanced, recurrent or metastatic esophageal squamous cell carcinoma (ESCC)	2020
			Malignant pleural mesothelioma	
			Metastatic or recurrent non-small cell lung cancer	
			Advanced or metastatic gastric cancer, gastroesophageal junction cancer, and esophageal adenocarcinoma	2021
			Esophageal or gastroesophageal junction (GEJ) cancer	
		Cemiplimab	Metastatic cutaneous squamous cell carcinoma (CSCC)	2018
			Locally advanced basal cell carcinoma (laBCC)	2021
			Advanced non-small cell lung cancer (NSCLC)	
	Anti-PD-L1	Atezolizumab	Advanced or metastatic urothelial carcinoma	2016
			Non-small cell lung cancer (NSCLC)	
			Triple-negative breast cancer(TNBC)	2018
			Small cell lung cancer(SCLC)	2019
			Melanoma	2020
			Hepatocellular carcinoma(HCC)	
		Avelumab	Metastatic Merkel cell carcinoma(MCC)	2017
			Advanced or metastatic urothelial cell carcinoma	
			Advanced renal cell carcinoma(RCC)	2019
		Durvalumab	Urothelial cell carcinoma	2017
			Non-small cell lung cancer (NSCLC)	2018
			Extensive-stage small cell lung cancer (ES-SCLC)	2020
Cytokines modulation	Interferon alfa-2b,recombinant	Intron A	Hairy cell leukaemia	1986
			AIDS-related Kaposi sarcoma	1988
			Melanoma	1995
			Follicular lymphoma	1997
	Interferon alfa-2a,recombinant	Roferon-A	Hairy cell leukaemia	1986
			AIDS-related Kaposi sarcoma	1988
			Chronic myelogenous leukaemia	1997
	Interleukin-2,recombinant	Aldesleukin	Melanoma	1998
			Renal cell carcinoma	1992
	Stimulates TNF, IL-12 and IFNγ	Imiquimod	Basal cell carcinoma	2004
CAR T-cell therapy	CD19-directed	Tisagenlecleucel	B-cell precursor acute lymphoblastic leukemia (ALL)	2017
			Large B-cell lymphoma	2018
		Axicabtagene ciloleucel	Large B-cell lymphoma	2017
			Relapsed or refractory follicular lymphoma (FL)	2021
		Brexucabtagene Autoleucel	Mantle cell lymphoma (MCL)	2020
		Lisocabtagene maraleucel	Diffuse large B-cell lymphoma (DLBCL)	2021
	B-cell maturation antigen (BCMA)-directed	Idecabtagene vicleucel	Relapsed or refractory multiple myeloma	2021
Vaccines	Autologous APCs with recombinant human PAPGM-CSF	Sipuleucel-T	Prostate cancer	2010
Oncolytic viruses	Genetically modified HSV-1 designed to replicate within tumours and produce GM-CSF	Talimogene laherparepvec	Melanoma	2015
Bispecific antibodies	CD19 and CD3 bispecific antibody	Blinatumomab	B cell acute lymphocytic leukaemia	2014
		Amivantamab-vmjw	Advanced or metastatic non-small cell lung cancer (NSCLC)	2021

## DNA Damage Repair Pathways

**Figure 1:**
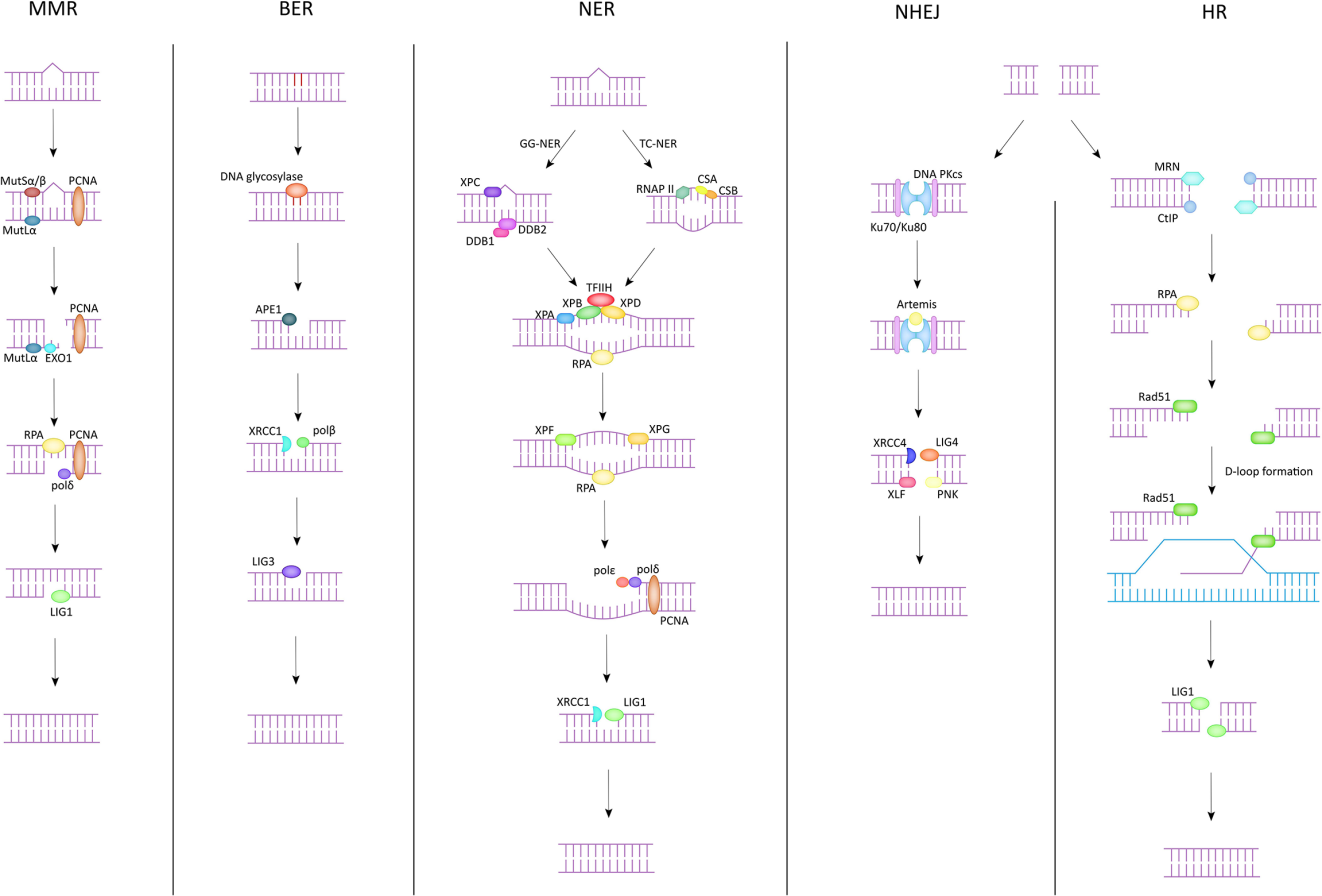
DNA damage repair pathway.

There are five common DNA damage repair pathways that play a key role in maintaining genome stability, and they are briefly described below (**Figure 1**).

### Mismatch Repair (MMR)

The main targets of MMR in the correction of DNA damage are base-base mismatches and insertion/deletion mispairs. Essential proteins in the MMR process, to which MutSα and MutSβ are sensitive to these misconfigurations (15). MutSα (MSH2-MSH6) recognizes mismatches and smaller nucleotide alterations and functions as a molecular switch after identifying the repair pathway to be initiated. MutSβ (MSH2-MSH3) is responsible for detecting larger nucleotide insertion/deletion and bends the DNA double helix after damage is detected, providing the basis for subsequent repair (16). During the execution of MMR, MutSα/MutSβ is the first to spot the damage areas and recruit at the damage sites. In the meantime, MutLα is assigned to exert endonuclease activity to cleave the single strand of DNA at the mismatch site. Subsequently, PCNA, EXO1 and other proteins act on the site to separate the mismatched part from the DNA strand. Finally, polymerase δ and DNA ligase 1 (LIG1) function together to fill the gap that appears after the trimming, completing the MMR process (17).

### Base Excision Repair (BER)

The BER pathway is often used to correct the deamidation and alkylation of bases in DNA molecules. The core component of the BER pathway is DNA glycosylase, which can be divided into two categories according to their functions (18). One class is monofunctional DNA glycosylases, such as UNG, MBD4, MYH, etc. The other has both 3’AP lyase activity and glycosylase activity, and the common ones are OGG1, NTH1, etc. (19) The DNA glycosylase then catalyzes the cleavage of the damaged site, exposing an AP site. Apurinic/apyrimidinic endonuclease 1 (APE1) cis-activates the DNA strand at the location of the AP site, yielding an independent 3’-hydroxyl fragment of DNA (16). This fragment will serve as a template to guide XRCC1 and DNA polβ to reinsert the missing bases and complete the filling of the processed deletion site (20). Finally, the break site is ligated by DNA ligase 3 (LIG3), and the whole BER process is completed (21).

### Nucleotide Excision Repair (NER)

NER is the only one among all DNA damage repair pathways that can correct UV-photolesions. In addition, NER also plays an important role in the repair of reactive oxygen species (ROS) induced base alterations, and intrastrand crosslinks (22). NER has been divided into two pathways depending on the target audience. One is global genomic repair (GG-NER), which plays a role in correcting genome-wide errors. The other is TC-NER, which only targets errors in the transcriptional strand of active genes for repair. These two are different in the early stage but are consistent in the late stage (23). In GG-NER, XPC and UV-DDB are the key genes in the process, DDB can be divided into DDB1 and DDB2 (XPE), and in general, XPC can directly identify DNA molecules with abnormal helix structure (24). However, there exist some complex situations that do not support correction in the above manner. Such cases would require that the damaged site is first identified by DDB2 and subsequently DDB1 is recruited and forms a complex structure with it. XPC can identify the DDB complex anchored to the DNA double helix and through this process complete the exclusion of the abnormal helix and the recruitment of TFIIH. The TC-NER pathway does not use the above-mentioned genes to exclude the distorted helix, but rather RNAP II, CSB, and CSA to initiate the error site recognition mechanism. The next link in both pathways is the same. Both direct the XPA to verify the site of injury and then complete the recruitment of replication protein A (RPA) (25). The role of RPA lies in its ability to bind to the template strand, which is ssDNA complementary to the damaged DNA strand, to keep it free from interference by other related factors and to ensure the accuracy of genetic information (26). Immediately afterwards, TFIIH forms a complex with XPB, XPD to unwind the helical structure at the wrong site (27). Subsequently, XPF and XPG incise the 5’ and 3’ single/double strand junction respectively. Meanwhile, PCNA, polδ, and polϵ are responsible for reconstructing the correct DNA fragment based on the template strand and filling it in the deletion. Finally, the scattered DNA fragments are connected by LIG1/XRCC1-ligase3 to restore the integrity of the DNA structure (28).

### Non-Homologous End Joining (NHEJ)

DSB is one of the most serious types of DNA damage, because it involves a wider region and more damaged fragments, which is more likely to cause damage to genomic stability and enhance susceptibility to many diseases. NHEJ and HR are two common DNA damage repair pathways that target DSBs. NHEJ can function in any process of the cell cycle, thanks to its repair independent of homologous sequences. DNA double-strand breaks are initially recognized by Ku70 (XRCC6)/Ku80 (XRCC5), which binds to the exposed broken ends after damage. This process has several important functions. First, Ku70/Ku80 binds to the break end and protects it from damage by other related enzymes. Second, this process provides the conditions for subsequent anchoring of DNA PKcs, which has a low affinity for the DNA duplex and can reach more than one hundred times the original affinity in the presence of Ku70/Ku80. Furthermore, the binding of Ku70/Ku80 to the broken ends can also improve the binding ability of XRCC4, LIG4, etc. to DNA ends, laying the foundation for the final processing (29). After Ku70/Ku80 binds to DNA broken ends, DNA PKcs interacts with Ku70/Ku80 to form a complex and complete anchoring (30). At the same time, Artemis is activated and given the ability to cut the DNA strand (29). Next, the DNA ends are treated by Artemis by endo-nucleation and XRCC4 activates LIG4 and forms a complex with it to act on the treated broken ends. The ligation of the DNA strand is done with the help of XLF, PNK and other substances. The NHEJ pathway is efficient and convenient for repairing damaged DNA double strands in a short period of time. Unfortunately, this approach is not precise enough, and the repair process is prone to fragment deletion and incorrect insertion.

### Homologous Recombination (HR)

The HR pathway is another repair pathway for DSBs besides NHEJ. Compared with NHEJ, the HR pathway can make the genome more precise and even identical to that before the injury. However, in contrast, the HR pathway exists only in S and G2 phases because sister chromatid must be present in the HR process to provide the homologous sequences necessary for repair. First, the damaged DNA ends are recognized by the MRE11-RAD50-NBS1 (MRN) complex, and then the damaged DNA segments are excised along the 5’ to 3’ direction in association with the C-terminal interacting protein (CtIP) to form single-stranded DNA (31). After this, BLM, EXO1 work together to provide the conditions for RPA anchoring on ssDNA, and the binding of RPA to ssDNA both replaces the faulty part of the structure and ensures the conformational stability of the exposed part of ssDNA. After this process is completed, Rad51, the most important protein in the HR pathway, will play a role in the replacement of RPA with the help of proteins such as BRCA2, Rad52, paralogs of Rad51 (32) and search for a template homologous to the damaged segment on the sister chromatid, which is also known as D-loop formation, which is crucial for the HR pathway to ensure accurate repair. Finally, DNA polymerase is used to synthesize a new DNA fragment based on the selected template, and LIG1 completes the ligation of the newly synthesized fragment to the initial fragment to repair the damaged DNA double strand (33).

## DNA Damage Repair Is Related to Innate Immunity and Tumor Immune Escape

**Figure 2:**
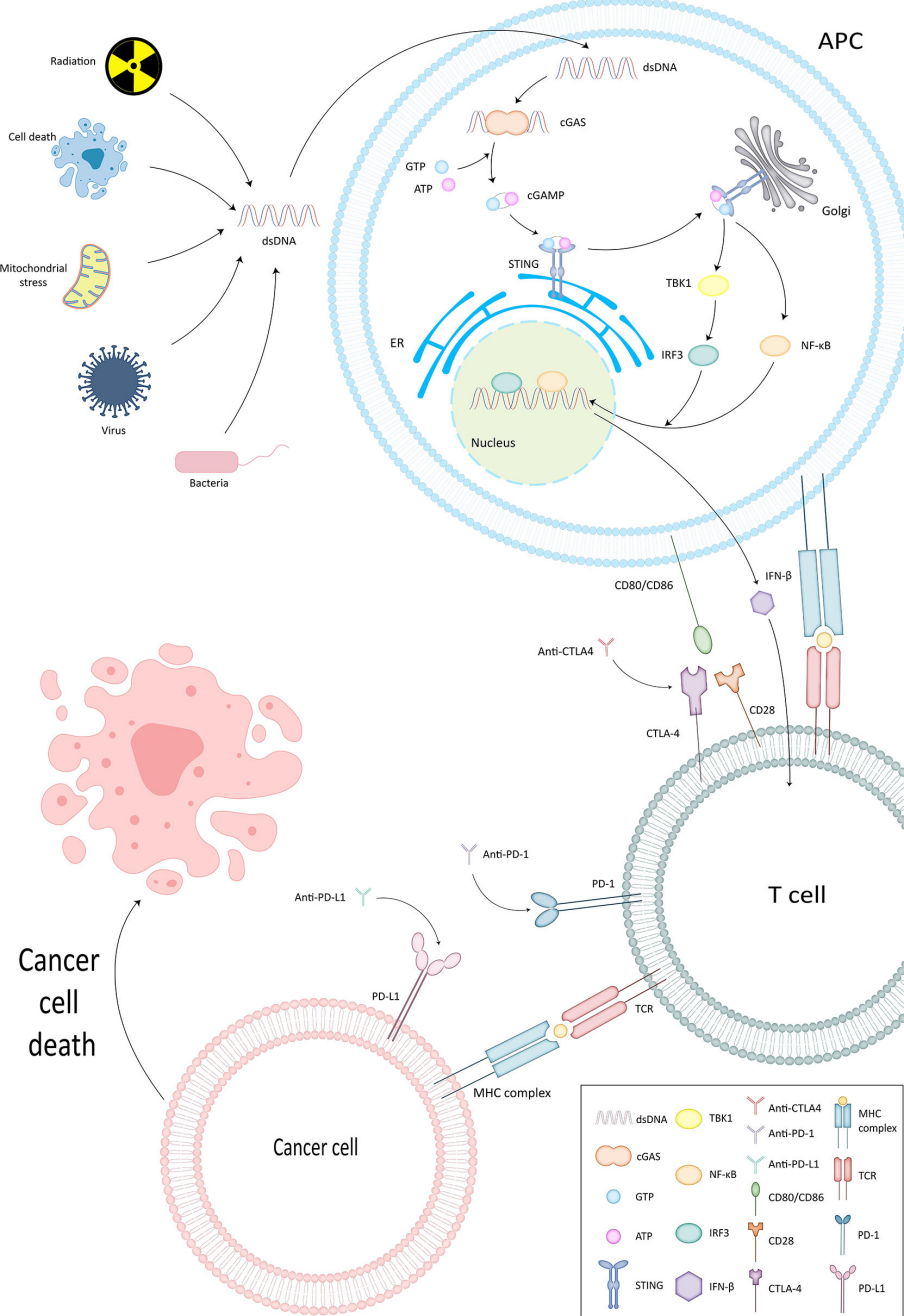
cGAS-STING pathway and ICI mechanism of action. In cGAS-STING pathway, cGAS binds to dsDNA and is subsequently activated to produce cGAMP. The latter interacts and activates STING on the endoplasmic reticulum membrane. STING then further confers TBK1 activity and sets the stage for TBK1 phosphorylation of IRF3, thus completing the recruitment of TBK1 and IRF3 by STING. Type I IFN is generated under the influence of IRF3 and functions to activate the immune system.

In **Table 1**, we have listed several methods currently approved by the FDA for tumor immunotherapy. Among them, immune checkpoint inhibitors (ICI) have been shown to have powerful immunotherapeutic activity. The role of DNA damage repair in the process of innate immunity has been confirmed. Next, since this section has already been reviewed by researchers, we will briefly describe important pathways in the immune response, explain the role of DNA damage repair in the process of tumor immune escape, and further analyze the mechanism of ICI targeting PD-1/PD-L1 (**Figure 2**).

### cGAS-STING Pathway

STING enables surveillance of tumor cells and participates in the innate anti-tumor immune process by inducing apoptosis. cGAS generates cGAMP that can interact with STING by binding to dsDNA (34). It also confers the ability to phosphorylate IRF3 by TBK1, thus completing the recruitment of TBK1 and IRF3 (35). Subsequently Type I IFN, which has an activating effect on the immune system (36, 37), is produced and exerts direct and indirect antitumor effects by promoting the production of perforin and granzyme by CTL and NK cells (10, 38, 39).

### Association of PD-1 and PD-L1 With Tumor Immune Escape

PD-1 is mainly expressed on activated T cells, B cells and macrophages (40). T cells can gain the ability to eliminate tumor cells by expressing PD-1 (41). However, tumor cells and APCs can express PD-L1, the ligand of PD-1 (42, 43) (**Figure 1**). By binding to PD-1, TIL apoptosis is induced on the one hand and CD4+ differentiation into regulatory cells (Treg) is stimulated on the other hand (44, 45). Weakening the recognition of tumor cells by T cells (46) and completing the immune escape (47). Anti-PD-1/PD-L1 destroys this process to wake up the immune response and prevent tumor cells from escaping.

## Targeted DNA Damage Repair to Explore Immunotherapy Biomarker

Glioblastoma (GBM) in brain tumors, one of the most lethal solid tumors, has a median survival rate of only 12-15 months despite various therapeutic modalities such as surgical resection, radiation therapy chemotherapy, and others have been carried out (48). Aggressive tumors like GBM possess cancer-resistant stem cells (CSCs) with a high capacity for self-renewal. The ability to acquire such strong self-healing characteristics is mainly due to the outstanding DNA damage repair capacity of GBM cells. Then, it is possible to increase the immunotherapy of brain tumors by disrupting the DNA damage repair process of brain tumors. And to identify cells or factors that may have a directive effect on TME, and use them as biomarkers to assess the feasibility and predict the effect of immunotherapy. Therefore, we tried to find biomarkers that have been applied or have potential value in the DNA damage repair pathway to provide guidance for the development of immunotherapy in brain tumors.

### Approved Biomarkers

#### GSCs

Glioma stem cells (GSCs) are a highly treatment-resistant population of GBM microenvironment components that assume a crucial role in tumor initiation, progression, and recurrence (49). During the early stages of tumorigenesis, GSCs have a major role in maintaining the reproductive potential of tumor cells. For example, the overexpression of transcription factors (TFs), Oct4 and Sox2 in GBM has been reported to promote tumor proliferation (50). In addition to this. Compared to the rest of the cells, a higher number of PD-L1 and immunosuppressive factors could be detected on the surface of GSCs, while the degree of CD80/CD86 expression was reduced (51). Moreover, GSCs interact with immune cells both to inhibit the antitumor effects of immune cells in the aforementioned manner (52), and to obscure their recognition by T cells through passive downregulation of MHC-I and antigen processing mechanisms to complete the immune escape process (53, 54). Khosravi et al. also reported similar results that GSCs can help tumor cells escape from the recognition correction of the immune system by upregulating specific immunosuppressive factors (55). Gangoso E et al. have further deepened their understanding. They believe that no matter what kind of treatment, when GSCs are discovered by the immune system, their DNA methylation and transcription processes will respond accordingly, and help them by secreting more chemokines. The DNA methylation and transcriptional processes of GSCs that survive are preserved, completing the process of downward transmission of immune evasion ability (56). In addition, the recruitment of macrophages, Tregs and myeloid-derived suppressor cells (MDSC) in the microenvironment is also inextricably linked to GSCs (57, 58). From this point of view, GSCs is a key biomarker for glioma immunotherapy, and some oncolytic viruses (OVs) targeting the effects of GSCs have been deeply studied. The concept of OVs is to selectively kill tumor cells without accidentally injuring normal cells. This is manifested by weakening or eliminating virulence factors so that they do not pose a threat to the growth and reproduction of normal cells. However, the virus itself retains the ability to fight against tumor cells, which is the most important basis for its function. Additionally, the OVs have the ability to recruit immune cells, that can continue the removal of tumor cells through the immune pathway (59, 60). Zika virus (ZIKV) is a typical example (61). ZIKV acts on the basis of GSCs, to which it is extremely sensitive, and can inhibit the proliferation of glioma cells by inducing apoptosis of GSCs, break the supporting effect of GSCs on immune escape, and further activate CD8+ T cells to complete the clearance of tumor cells (62). Also, because of the high enrichment of integrins αVβ3/αVβ5 in GSCs, OVs can target GSCs by acting on integrins (63). It has also been demonstrated that GSCs achieve tumor cell invasion and metastasis by affecting the extracellular matrix (64). Thus, GSCs and therapies targeting GSCs have become the focus of immunotherapy for brain tumors, and the use of GSCs as biomarkers may provide a major reference for the treatment of brain tumors.

#### TAMs

Tumor-associated macrophages (TAMs) are the most abundant cells in the GBM microenvironment, accounting for roughly 30%-50% of all cells in TME (65). TAMs strictly include both microglias and macrophages of embryonic or bone marrow origin. There are numerous literature TAMs have been reported to have immunosuppressive effects on TME (66, 67). TAMs can be stimulated by a variety of cytokines and thus activate differentiation potential into M1-type TAMs, which have suppressive effects on tumor cells, and M2-type TAMs, which promote tumor cell proliferation and metastasis (68, 69). Lipopolysaccharide and IFN-γ, etc. can induce the differentiation of TAMs to M1-type and endow M1-type TAMs with the function of secreting pro-inflammatory cytokines and activating other immune cells, such as natural killer cells (NK) and dendritic cells (DC). M1-type TAMs can both directly induce tumor cell death through their own cytotoxic effects and indirectly serve anti-tumor purposes by phagocytosis, which enables the process of neoantigen presentation on the surface of tumor cells and provides conditions for adaptive immunity to proceed (70). Both have prominent contributions to probing tumor cells and slowing their proliferation. In contrast, M2-type TAMs are strongly associated with the malignant biological behavior of GBM cells (71). M2-type TAMs can be activated by a variety of cytokines, such as interleukin-4 (IL-4), colony-stimulating factor-1 (CSF-1) and tumor growth factor-β (TGF-β). These cytokines play different roles in the biological behavior of GBM cells. For example, tumor cells can achieve the recruitment of microglias by secreting CSF-1 and contribute to their differentiation to M2-type (72). And the function of TGF-β is highlighted in its provision of conditions for tumor cell metastasis (73). In addition, M2-type TAMs have remarkable ability in inducing GBM cytogenesis and immune escape, based on which they assume important functions in the process of tumor cell growth and metastasis. It has been reported that M2-type TAMs contribute to the vascular proliferation of GBM cells by releasing insulin-like growth factor binding protein 1 (IGFBP1) and IL-6 to contribute to the expansion of tumor blood vessels in TME and the enhancement of blood supply to tumor tissues (74). Meanwhile, M2-type TAMs can also secrete IL-10 through the JAK2/STAT3 signaling pathway to induce GBM cell genesis and promote tumor progression (75). Not only that, M2-type TAMs also have the ability to recruit Treg, and the number of Treg in the microenvironment shows a positive correlation with poor prognosis during tumor cell progression (70). As well as the inhibition of CTL cells in TME, which are responsible for the poor response of GBM to immunotherapy based on T-cell principles (70). With the increasing attention to the field involving TAMs, there are more and more reports about the involvement of M2-type TAMs in tissue remodeling, induction of hypersensitivity responses, domination of immune microenvironment formation, and promotion of GBM progression (76–79). TAMs are gradually becoming key targets in the fight against GBM (80, 81). Therefore, there are now a number of therapies targeting TAMs that are continuously being investigated. Basically, they are all based on the principle of blocking the migration of monocytes into TME and inhibiting the production of M2-type TAMs. Lee, Chanhee et al. found that artificially interfering with TAMs to convert them to M1-type and preventing them from converting to M2-type could inhibit the proliferation of GBM (82). This provides a new possibility for controlling GBM growth and attenuating GBM resistance to immunotherapy, and may provide a guiding role in the treatment of GBM.

#### ATM

The ATM gene encodes a protein that is an important cell cycle checkpoint kinase that phosphorylates important sites such as CHK2, p53, MRN complex and plays a prominent role in the HR pathway (83). ATM, an important factor in DNA damage repair, signals in association with PARP-1 to activate E3 ubiquitin ligase within one hour of recognition of a DNA double-strand break. And further recruit STING to achieve activation of nuclear factor NF-κB. Activated NF-κB has been shown to play an important role in tumor progression and clearance of tumors by the immune system (41, 84). Wang L et al. came to a similar conclusion that the ATM-CHK2 axis could be a potential target during tumor therapy and that selective inhibition of the checkpoint signaling axis ATM-CHK2 could activate the body’s intrinsic innate immunity and enhance ICI therapeutic efficacy, which has been demonstrated in ARID1A-deficient tumors (85). In addition, Sato H et al. first elucidated that DNA double-strand breaks can upregulate PD-L1 expression in an ATM-dependent manner (86). Hu et al. demonstrated that ATM inhibition can activate the cGAS-STING pathway by promoting cytoplasmic leakage of mitochondrial DNA and downregulating mitochondrial transcription factor A (TFAM) and in this way enhance the effect of ICI treatment. Therefore, mutations in ATM can be used to predict the clinical efficacy of ICI as an ICI therapeutic target and biomarker (87). In addition, ATM has also been reported to achieve enhancement of ICI therapeutic efficacy through the cGAS-STING-independent pathway (88). In addition, there is the most important point for brain tumors. ATM/ATR has been shown to be an important cause of chemotherapeutic drug resistance in GBM tumors, so could the detoxification of resistance to chemotherapeutic drugs as well as upregulation of immunotherapy sensitivity be achieved by blocking ATM/ATR. Meanwhile, the application of TMZ resulted in replication fork arrest of GBM cells and successive activation of ATR-Chk1 axis and ATM-Chk2 axis, which may be potential targets for combining chemotherapy with immunotherapy (89).

#### CTLA-4

CTLA-4 is a coinhibitory molecule expressed by Tregs cells that can be regulated by activated CD4+ versus CD8+ T cells and achieve interference with T-cell activation (90, 91). CD28 is expressed by activated CD4+ versus CD8+ and has a role in promoting immune responses and activating T cells. When the T-cell receptor (TCR) is engaged, CTLA-4 increases rapidly at the immune synapse through cytoskeletal reorganization, achieving enrichment. The CTLA-4 that reaches the synaptic site has a higher affinity for CD80 (B7-1) and CD86 (B7-2) than CD28 (92, 93). The above process is extracellular pathway. In addition, CTLA-4 has been shown to function through intracellular pathways, specifically by binding to CD80/CD86 and acting within activated conventional T-cells. CTLA-4 suppresses immune responses by recruiting SHP-2 and PP2A to immune synapses (92, 94). Different routes to the same goal, both of which negatively regulate T-cell proliferation (95, 96). Ensure T-cell activity by fine-tuning the TCR to help safeguard beneficial anti-pathogen and anti-tumor responses, while maintaining tissue integrity, promoting tissue repair, and regulating immune sensitivity (97).

### Other Potential Biomarkers

RPA is a ssDNA binding protein, which plays an important role in both NER and HR (98, 99). In the primary GBM cell lines, high-level expression of RPA was detected. RPA expression can also be detected in differentiated GBM cells, and it has been observed that RPA70 and RPA14 have priority in expression. RPA mediates the high invasiveness of GBM. It is believed that blocking the function of RPA may increase the responsiveness of radiotherapy. And RPA can function by interacting with sites such as ATR, Rad51, BRCA1/2, and p53 (100, 101). This may be a potential biomarker for highly aggressive tumors such as GBM (102).

Zhang J et al. supplied a new immune target where DNA-PKcs could influence TGF-β1, a significant factor in the epithelial-mesenchymal transition (EMT) process (103). DNA-PKcs deletion enhances ICI treatment (104). The mutant group had a more satisfactory survival outcome after ICI treatment compared to patients with normal DNA-PKcs (105). Echoing this, Yang H et al. concluded that the combination of immunotherapy with DNA-PKcs as a target can have a synergistic effect, with the result that patients can benefit more from immunotherapy (106). There is also a DNA-PK inhibitor called CC-115, which is able to cross the BBB, whose efficacy in patients with GBM is being tested and CC-115 is being considered in combination with radiotherapy and TMZ for GBM (107).

In recent years, there has also been significant progress in the exploration of protein-coding RNAs, and it is thought that RNA dysfunction can also influence cancer development (108). Subsequently, immunotherapies targeting RNAs have also emerged, particularly LncRNA, whose expression has been shown to be associated with various immune checkpoints such as PD-1/PD-L1 and CTLA-4 (109). It has also been shown that Lnc-talc interferes with the action of temozolomide by affecting the immune microenvironment, leading to GBM resistance (110, 111). In addition to this, epidermal growth factor receptor (EGFR) and mesenchymal-epithelial transition factor (MET) signaling pathways have been demonstrated in temozolomide resistance (112). The experiments conducted for the above new targets provide new therapeutic strategies for GBM and offer the possibility to explore immunotherapy for GBM.

## Combined DNA Damage Repair-Based Therapy With Immunotherapy

A growing number of experiments have shown that therapies that destabilize DNA and disrupt the repair process of tumor cell damage, can reshape the tumor immune microenvironment and offer the possibility of applying immunotherapy to tumor cells that are resistant or less sensitive to immunotherapy. This is particularly important for the application of immunotherapy in brain tumors. On this basis, it becomes feasible and meaningful to combine immunotherapy with other tumor treatment modalities.

### Radiotherapy With Immunotherapy

It is well known that radiation therapy corrects tumor mainly through two mechanisms, one is the production of free radicals, which induce oxidative responses in the body and activate damage-associated molecular patterns (DAMPs), and in this way enhance DC function. The other is DNA damage, the vast majority of which is DNA strand breaks, with double-strand breaks (DSBs) being the key damage in radiation-killed cells. The role that radiation therapy plays in the immune process has been discovered step by step in recent years. Its effect on immune response is mainly to enhance tumor immunogenicity by increasing tumor-recognizable neoantigens, expression of MHC molecules, etc. (113) And to recruit CD8+ T cells and DC cells to awaken the body’ s anti-tumor immune process (114). Radiotherapy triggers immunogenic cell death by both of these means (115), enhances DC function and further promotes antigenic expression of dendritic cells (DCs) (116), allows DCs to be recruited between tumor tissues. Common DAMPs include HMGB1 and calreticulin, etc. (117) HMGB1 allows DC cells to acquire the ability to process and present antigens by acting on toll-like receptor (TLR) 4 receptors and furthermore induce T cell immune responses (118). Cells expressing calreticulin on the surface can be recognized by DCs and achieve phagocytosis of would-be dead cells. In contrast, DSBs resulting from DNA damage after radiotherapy produce cytosolic DNA fragments and micronuclei that activate the cGAS/STING pathway (119, 120). Further stimulates the production of IFN, increases the expression of MHC molecules on the surface of tumor cells, promotes the maturation of DC cells and drives other immune-related processes (111, 121–124). To achieve the purpose of enhancing the recognition ability of the immune system. Because of this, radiotherapy is also thought to be involved in the DNA damage repair process known as activate mTOR signaling (125). However, the effects of radiotherapy go far beyond this. Radiotherapy has also been reported to induce the expression of adhesion molecules ICAM and VCAM1 on tumors and endothelial cells, and in this way block the binding of adhesion molecules to T cells. Then reduced the incidence of tumor rejection (126). In addition, radiotherapy has been shown to increase the expression of inflammatory chemokines, such as CXCL5 and CXCL2, which recruit suppressive cells in TME while producing TGFβ and participating in the tumor immune process (126, 127). The common outcome of these aforementioned post-radiotherapy alterations is to promote the infiltration of CD8+ T cells in TME and to achieve up-regulation of PD-L1 expression by all of the above (128). Therefore, this behavior is a key part of the therapeutic effect of radiotherapy combined with PD-1/PD-L1 inhibitors (129). More importantly, the increase of MDSCs in TME after radiotherapy, which has been shown to drive tumor growth and angiogenesis, upregulate CTLA-4 expression in Tregs (130) and inhibit cytotoxic T-cell activation acting as a coordinating immunosuppressive agent (131). Furthermore, it has been suggested that TAMs and regulatory T-cells (Tregs) are more resistant to radiation than T cells, contributing to the enrichment in TME after radiotherapy (132). Radiotherapy can also activate NK cell action by upregulating the NKG2D receptor in the co-stimulatory receptor, achieving immunomodulation by interfering with the innate immune system. Also, radiotherapy has been reported to act on TNF receptors (133). Including FasL, TNF-α receptor, TRAIL-R1 and TRAIL-R2. Since CTL can express ligands of the above receptors, it makes tumor cells more sensitive to the action of CTL after radiotherapy. This also explains from a certain perspective, why some tumors gradually show a more satisfactory sensitivity to immunotherapy after receiving radiotherapy. It may be related to the direct or indirect recruitment and activation of CTL caused after radiotherapy. For example, IFN-γ and TNF-α produced after radiotherapy can induce CXCL9, CXCL16, etc. And then further collect CTL into TME. These reports support that the effect of radiotherapy on DNA damage repair can act on the immune system and provide a theoretical basis for the feasibility of radiotherapy combined with immunotherapy (134). Not only that, there are many animal experiments embarking on the combination of radiotherapy and immunotherapy.

Radiation therapy (RT) is widely used in patients with solid tumors, and GBM is no exception. Several preclinical studies have reported that RT can lay the foundation for immunotherapy and improve the response rate of immunotherapy. The most widely used immunotherapies are ICIs, and three types of ICIs have been approved by the FDA for clinical use, namely anti-CTLA-4, anti-PD-1 and anti-PD-L1. TIM-3 is a negative regulator that is widely expressed on Tregs and NK cells, and blocking TIM-3 can help CD4/CD8+ T cells to restore specific immune function, which is helpful in relieving the immune resistance of tumor cells (135). It has been shown that TIM-3 expression is elevated in glioma patients and can be detected not only on the surface of TIL, but also in circulating blood lymphocytes (136, 137). Kim, Jennifer E et al. attempted to apply anti-TIM-3 along with stereotatic radiosurgery (SRS) and analyzed the role of the combination in the treatment of glioma. The experiment showed that the median survival time of SRS alone was 27 days, while the combined anti-TIM-3 group could extend up to 100 days. Also, the team experimented with the possibility of combining two ICIs, anti-PD-1 and anti-TIM3. It was shown that both PD-1 and TIM-3 have inhibitory effects on the secretion of some cytokines such as IFNγ and TNFα (138). Compared to anti-PD-1 alone, Kim, Jennifer E et al. did not observe an increase in the amount of IFNγ in the combination of anti-PD-1 with anti-TIM-3. However, there was positive feedback from the trinity treatment approach of both ICI combined with SRS compared to the anti-PD-1 combined with SRS group. That is, a trend was detected to promote the secretion of IFNγ, TNFα, and the secretion of such cytokines was widely shown to be associated with prolongation of OS. Although the combination of ICI and RT has been repeatedly reported to have a synergistic effect in saving brain tumors, there are conflicting opinions about the side effects (139). Clausi MG et al. suggested that RT in combination with ICI induced activation of CD8+ T cells and polarization of TAMs. It was reported that CD8+ T cells were not found in normal brain tissue, while traces of CD8+ T cells were detected in the white matter and hippocampus of mice with brain tumors after combined treatment. Meanwhile, in studying the side effects of the combination treatment, they evaluated the cognitive and behavioral performance and neuroinflammation in the mice. The results showed that although the combination therapy reflected better results on tumor control, it also induced cognitive and behavioral, neuroinflammatory and other side effects that affected the quality of survival (140). However, positive side effects of the combination treatment have also been reported, as Qiu B et al. found that RT combined with ICI treatment can lead to permanent depletion of neuroblasts in the subgranular zone (SGZ) of the hippocampal dentate gyrus, which can indirectly protect the function of the hippocampal region. The addition of anti-PD-1 provided a cerebral protective effect relative to RT applied alone (141).

### Chemotherapy With Immunotherapy

Chemotherapy is one of the most effective methods of treating tumors. It is used to kill tumor cells through the application of drugs to achieve a therapeutic goal. One of the main advantages of chemotherapy over traditional surgery and radiation therapy is that chemotherapy is a systemic treatment. Chemotherapy drugs can act on most tissues throughout the body through blood circulation, which is an outstanding advantage in treating metastatic cancer. Also for GBM, the application of chemotherapeutic drugs has shown to be extremely superior, and TMZ plays an integral role in GBM treatment. However, in recent years, as chemotherapy has become more widely understood, it has been found that chemotherapy has not only cell-damaging effects, but also significant effects on the immune system. Chemotherapy can expose neoantigens on the surface of tumor cells, which are recognized by DCs and presented to CTL cells, further activating the antitumor immune response. In recent years, the exploration of the relationship between chemotherapy and immune response has gradually become a popular topic. Toll-like receptors (TLRs) mentioned before is one of the cases. Many immune cells activate the relevant immune response through the interaction between receptors called TLRs and pathogens (142). Based on this, molecules that have agonistic effects on TLRs have won widespread attention. One of them is an oligonucleotide called CpG-ODN (143). CpG-ODN is classified as A, B and C according to the type of cells it acts on. Among them, type B CpG-ODN has been shown to inhibit tumor cell growth by acting on TLR9 in several preclinical models, with long-lasting effects and immune memory detected in specific subjects (144). TLR4 has also been found to be induced by paclitaxel and cause immune cell death. But chemotherapy is also thought to activate the immunostimulatory pathway and can be used to kill cancer cells by this mechanism. After the application of drugs such as docetaxel, oxaliplatin and cyclophosphamide (CPA). ATP, HMGB1 and tumor cell surface calcineurin can be detected. These exposed neoantigens increase the possibility of tumor cells being recognized by immune cells (145, 146). Chemotherapeutic agents can also directly modulate immune cell populations. Cyclophosphamide depletes immunosuppressive myeloid suppressor cells and Treg cells, relieves their inhibitory effects on NK and T cells, elevates the innate immune response, and promotes Th1 cytokine production (147). Cyclophosphamide has been used as a first-line chemotherapeutic agent for many years, and although it has been shown to have excellent anti-tumor performance, experimental feedback suggests that combination therapy can achieve superior results compared to monotherapy. Jordan, M et al. conducted a study in 2016, which showed that the addition of CpG-1826 immunotherapy on day 12 after the application of cyclophosphamide therapy prolonged the duration of immune response prompted by cyclophosphamide and a satisfactory antitumor response was observed. Combining CpG-1826 after cyclophosphamide treatment minimizes the ablation of immune cells by cyclophosphamide and prolongs the duration of response (142). It is well known that temozolomide synchronized chemotherapy is an indispensable and critical part of GBM treatment, so how to use TMZ rationally to enhance the effect of GBM immunotherapy is being paid attention to (148). Although reduction of multiple lymphocytes in TME can be observed after TMZ application, in some cases, TMZ was found to induce anti-tumor immune response (149). Hasan, Md Nabiul et al. focused on the effect of Na/H exchanger 1 in combination with TMZ on immunotherapy. They found that NHE1 was closely related to the immunosuppressive TME of GBM, which is one of the reasons for downregulating the sensitivity of GBM to the immune response (150). And further validated the effect of combination treatment of NHE1 inhibitor HOE642 with TMZ on PD-1. The results showed that the infiltration of GAMs and T cells was significantly increased in the combination treatment group, and Th1 was activated along with enhanced anti-tumor immunity. In addition to this, the combination therapy increased the sensitivity of anti-PD-1 treatment modality, providing the possibility of ICI in GBM treatment (151). The rationale for this therapeutic approach focuses on the metabolic reprogramming of TAMs and T cells that has been discovered in recent years, which plays an important value in the anti-GBM immune response. This is thought to be a pathway for tumor cells to evade surveillance by the immune system, i.e., by upregulating PD-L1 and binding to PD-1 on the surface of T cells to induce T cell apoptosis to complete the immune escape process (152). Although this process is a survival strategy for tumor cells, it also brings an idea for the application of immunotherapy, i.e., the possibility of targeting immune checkpoints for blockade, which is a breakthrough for brain tumors that are part of cold tumors. One trial specifically analyzed the effect of combining TMZ with ICI and reported that when TMZ was administered systemically, the combined ICI treatment did not show any survival advantage. In contrast, when TMZ was administered locally, the survival advantage appeared to be significantly altered with the combined application of ICI. This seems to indicate that the effect of TMZ on anti-tumor immune response is related to factors such as dose and route of administration (153). In addition, the oncolytic virus, which has been widely studied in brain tumors, has shown satisfactory results in joint experiments with TMZ. Sampson JH et al. demonstrated that an enhanced antitumor immune response was observed with the application of TMZ in GBM patients treated with oncolytic virus.

### DNA Damage Repair Inhibitors With Immunotherapy

#### Poly ADP-Ribose Polymerase Inhibitor (PARPi)

PARP, is a DNA repair protein. It maintains genomic stability by repairing damaged DNA single strands during DNA damage repair, PARP is mainly divided into PARP1 and PARP2, with PARP1 playing a key role in the efficient repair of DNA single strand breaks (SSBs) (154). This mechanism is also generalized in tumor cells. A large proportion of antitumor drugs interfere with the normal life activities of tumor cells by damaging their DNA, and in this way, they aim to kill them. Unfortunately, tumor cells can protect themselves through the above damage repair mechanism. Inhibitors of poly ADP-ribose polymerase (PARPi) have emerged as new tumor therapeutic agents (**Table 2**). PARPi applies the concept of synergistic lethality of DNA damage repair by competitively binding to PARP to inhibit HR, resulting in the accumulation of large amounts of single-stranded DNA in tumor cells that are not repaired in time (155). This enhances the efficacy of chemotherapeutic agents. However, PARPi’s contribution to tumor therapy goes far beyond this. While inhibiting DNA-SSB repair, PARPi acts with the cofactor NAD+ to anchor PARP1 to damaged DNA, forming a stable PARP-SSB complex. In turn, the replication fork disintegrates upon contact with the PARP-SSB complex and consequently leads to more severe DSBs, which serve to induce tumor cell death (154, 156). More notably, PARPi has a targeted effect on BRCA-deficient tumors. BRCA is also a DNA repair protein, but unlike PARP, BRCA is primarily responsible for the repair of DSBs. Therefore, BRCA1 and BRCA2-deficient tumor cells are very dependent on PARP for repair, and for this reason, PARPi has a significant effect on DNA damage in such tumor cells, showing great cytotoxicity (147, 157). Therefore, it has become a precision drug for cancers with DDR defects in the HR pathway and was approved by the FDA in 2018 for the treatment of BRCA-deficient cancers. Many studies have shown that the antitumor effect of PARPi is related to innate immune response in addition to the induction of DNA strand breaks (158). PARPi accomplishes antitumor efficacy by activating the cGAS-STING pathway, recruiting CD8+ T cells, and inducing type 1 interferon (IFN) signaling, thereby resetting or initiating the tumor microenvironment (159). Currently, most of the clinical trials assessing the role of PARP in GBM neglect to assess BRCA (160), which may be related to the low frequency of BRCA mutations in GBM (161). PARP expression has been reported to be associated with tumor grade as well as poorer survival (162). An increase in tumor radiotherapy sensitivity was observed in *in vitro* experiments applying PARPi against GBM models (163). With a better understanding of the molecular relationship between PARP and GBM (164), PARP may be used as a biomarker to assess prognosis and drug resistance mechanisms.

**Table 2:** FDA-approved PARPi class drugs.

PARP inhibitors	FDA-approved cancers	Year of approval
Olaparib	Advanced ovarian cancer	2014
	Primary peritoneal cancer	2017
	HER-2 negative metastatic breast cancer	2018
	Metastatic pancreatic adenocarcinoma	2019
	Metastatic castration-resistant prostate cancer(mCRPC)	2020
Rucaparib	Ovarian cancer	2016
	Recurrent epithelial ovarian	2018
	Primary peritoneal cancer	
	Metastatic castration-resistant prostate cancer(mCRPC)	2020
Niraparib	Primary peritoneal cancer	2017
	Advanced ovarian	2019
	Primary peritoneal cancer	
Talazoparib	HER-2 negative locally advanced or metastatic breast cancer	2018

#### Other DNA Damage Repair Inhibitors

Heat shock protein 70 (Hsp70) is often expressed on the surface of highly aggressive tumor cells such as GBM, while the upregulation of Hsp70 can also be observed after radiotherapy and chemotherapy (165). Shevtsov M et al. investigated the antitumor effect of a combination therapy consisting of Hsp70-peptide TKD/IL-2-activated NK cells and anti-PD-1 on GBM in mice. The results showed that both alone retarded the growth and migration of tumor cells and prolonged the OS of the GBM mouse model, while the combination therapy further improved the outcome parameters compared to the monotherapy modality. The OS in the combination treatment group was 2.3 times higher than that in the control group. Tumor tissue sections showed increased infiltration of CD8+ T cells and NK cells in the treatment groups, with the most pronounced immune cell infiltration in the combination treatment group and a 1.5-fold increase in anti-tumor cytotoxicity. This is consistent with previous reports that blocking PD-1 on the surface of NK cells can enhance immune responses (166).

In addition, phosphatases are also attracting attention as new brain tumor targets (167). It has been claimed that PP2A can activate CTLA-4 on the surface of T cells through dephosphorylation, which has a potential inhibitory effect on the immune function of CTL (168). Besides, researchers also found that the negative immune regulatory function of Treg was broken in the model of PP2A deficiency, which contributed to the proliferation of TIL (169). Based on this, Maggio D et al. considered to analyze the effect of simultaneously targeting both PP2A and PD-1 checkpoints on tumor control. It was found that simultaneous blockade of PP2A and PD1 significantly improved OS in GBM mice, with a substantial increase in the number of immunoreactive T cells in the group compared to the control group, and resulted in complete regression of GBM in about a quarter of the mice. The team further speculated that this might be related to the activation of the mTORC1 pathway after the application of PP2A inhibitor (170). Another molecule highly expressed in GBM is arginase (ARG), which is more easily detected especially in TAMs with highly aggressive GBM. Some studies have confirmed that ARG has a proliferative effect on CTL, while lower plasma concentrations of ARG are often accompanied by the appearance of significant immunosuppression (171). Inhibition of ARG restored the function of TAMs and NK cells and improved the sensitivity to anti-PD-1. Zhang J et al. applied an anti-ARG called OAT-1746 in combination with anti-PD-1 in a GBM mouse model, and OAT-1746 could penetrate the BBB, which is known for its defensive capabilities. The results observed an increase in the proportion of CD3+ T cells in the TME of mice after the combination treatment, which is important for considering the simultaneous inhibition of ARG and PD-1 in the GBM population for potential feasibility. Meanwhile, decreased expression of CCL2 and CCL7 was detected in the experimental group, and the expression of CCL2 is closely associated with tumor angiogenesis and high invasiveness (172), while CCL7 has a recruitment effect on Treg (173). The positive feedback of combination therapy for TME may be related to OAT-1746 affecting the expression of CCL2 and CCL7-related genes (174).

## Conclusion

Tumor immunotherapy has evolved rapidly in the past decade. And today, many therapies targeting the immune response have been approved and are used in clinical practice. However, unfortunately, the use of immunotherapy in the field of brain tumors still has not progressed much. Due to the existence of BBB, the intracranial system can protect the brain tissue from damage, but also increase the difficulty of drugs breaking through the barrier. Moreover, brain tumors are immunologically “cold tumors” that do not show satisfactory sensitivity to immunotherapy, and the use of autoimmune response to achieve clearance of brain tumors remains a major challenge. Fortunately, tumors are associated with genomic instability, and DNA damage repair is an important way for the body to maintain and correct genetic information. Therefore, targeting DNA damage repair mechanisms in tumor therapy may be a breakthrough guide in the fight against tumors. This study also focused on this hot topic and analyzed the intrinsic link between immunotherapy and DNA damage repair. We also found that appropriate biomarkers are particularly important for evaluating immunotherapy. TAMs, as the largest group of cells in brain tumor TME, play an important role in immune regulation of the microenvironment. And biomarkers such as MSI and ATM, which have been widely used in “hot tumors”, also seem to guide brain tumors that are not sensitive to immunotherapy. RPA, DNA-PKcs and other proteins in the DNA damage repair process may provide a precursor assessment for the rational implementation of immunotherapy in brain tumors and may serve as a guide for immunotherapy. At the same time, we discuss the feasibility of combining immunotherapy with other treatments. DNA double-strand breaks during radiation therapy, chemotherapy that partially targets DNA damage directly, and targeted therapies that are now used to break the DNA damage repair process in tumor cells all provide favorable premises for the use of immunotherapy. We also describe the current state of research in combination therapy. Even so, little is known about brain tumors and TME and how the promising immunotherapy can be used in the clinic. The application of immunotherapy in brain tumors remains a major challenge that needs to be explored jointly by clinicians, genomics, translational medicine and other multidisciplinary personnel. Together, we will reveal the intrinsic link between DNA damage repair processes and brain tumor immunotherapy, and provide inspiration and support for the application of damage repair in brain tumor immunotherapy.

## Author Contributions

SZ and BX wrote the article. WM and HC conducted cutting-edge searches and modifications. CJ provided critical perspectives on the article ideas. JC and XM participated in the figure design and article revision. All authors contributed to the article and approved the submitted version.

## Funding

This study was supported by 1. The National Natural Science Foundation of China (No. 81874204, No. 81772666, No. 81972817, No. 82073298, No. 82003022); 2. Excellent Young Talents Project of Central Government Supporting Local University Reform and Development Fund (0202-300011190006); 3. Karolinska Institute Research Foundation Grants 2020–2021 (No. FS-2020:0007); 4. The China Postdoctoral Science Foundation (2018M640305, 2019M660074, 2020T130157); 5. The Heilongjiang Postdoctoral Science Foundation (LBH-Z18103, LBH-Z19029); 6. The Research Project of the Health and Family Planning Commission of Heilongjiang Province (2019-102); 7. The Young and Middle-aged Innovative Scientific Research Fund of the Second Affiliated Hospital of Harbin Medical University (KYCX2018-08).

## Conflict of Interest

The authors declare that the research was conducted in the absence of any commercial or financial relationships that could be construed as a potential conflict of interest.

## Publisher’s Note

All claims expressed in this article are solely those of the authors and do not necessarily represent those of their affiliated organizations, or those of the publisher, the editors and the reviewers. Any product that may be evaluated in this article, or claim that may be made by its manufacturer, is not guaranteed or endorsed by the publisher.
